# The Homœopathic Journal of Obstetrics, Gynecology, and Pedology

**Published:** 1892-08

**Authors:** 


### THE HOMŒOPATHIC JOURNAL OF OBSTETRICS, GYNECOLOGY, AND PEDOLOGY, for July, contains discussions on nearly a score of topics, all of them of vital importance to every physician in general practice.

In pursuance of the purpose to give each issue of the *Journal* a character of its own, Dr. Winterburn devotes this entire number to the consideration of the diseases of children. As will be remembered, the May number of the *Journal* contained a notable symposium on the repair of the lacerated perineum, in which thirty prominent gynecologists and surgeons took part. The July issue is increased to 128 pages, and contains contributions from thirty-four prominent physicians, including all the papers in pedology read at the recent meeting of the American Institute of Homœopathy, at Washington, and six papers read before the New York Pedological Society, beside others. Dr. Winterburn also contributes about fourteen thousand words in the form of editorials, therapeutic hints in the management of diseases of children, book reviews, and an address delivered at Albany, last February, entitled "The First Hours of Life."

The leading article of this number is by Dr. Talcott, of the Middletown Asylum, on "The Insane Diathesis," in which he sets forth in glowingly eloquent words the causes of insanity.

Dr. Millie J. Chapman, in her bureau address on pedology, before the American Institute of Homœopathy, makes a study of three great H's—Hygiene, Heredity, and Homœopathy. President Nottingham, of the Michigan Homœopathic Medical Society, calls attention to the connection between croup, chronic enlargement of glands, and tuberculosis. This admirable thesis should be in the hands of every practitioner of medicine.

To mention only a few of the other good things in this number: Prof. Crank, of Cincinnati, discourses on coryza in childhood as a neurosis; Dr. Johnson, of Sullivan, Illinois, deserves special praise for his interesting paper on the care of infants; Dr. Helena M. Cady has a thoughtful little essay on "The Needs of the Baby;" Editor Van Baun, of the *Hahnemannian Monthly*, has a thoroughly practical article on pneumonia in children; Dr. Ripley, of Minneapolis, in "Some Overlooked Causes of Disease in Children," makes eloquent appeal against sexual vice; and Prof. Danforth, of New York, reports a case of acute parenchymatous nephritis in an infant six months old.

